# Algal Extracts for Green Synthesis of Zinc Oxide Nanoparticles: Promising Approach for Algae Bioremediation

**DOI:** 10.3390/ma16072819

**Published:** 2023-04-01

**Authors:** Ahmed E. Alprol, Abdallah Tageldein Mansour, Hossam S. El-Beltagi, Mohamed Ashour

**Affiliations:** 1 National Institute of Oceanography and Fisheries (NIOF), Cairo 11516, Egypt; 2 Animal and Fish Production Department, College of Agricultural and Food Sciences, King Faisal University, P.O. Box 420, Al Hofuf 31982, Saudi Arabia; 3 Fish and Animal Production Department, Faculty of Agriculture (Saba Basha), Alexandria University, Alexandria 21531, Egypt; 4 Agricultural Biotechnology Department, College of Agriculture and Food Sciences, King Faisal University, P.O. Box 420, Al-Ahsa 31982, Saudi Arabia; 5 Biochemistry Department, Faculty of Agriculture, Cairo University, Giza 12613, Egypt

**Keywords:** nanotechnology, ZnO-NPs, green synthesis, biological extracts, microalgae, seaweeds, aquatic plants, wastewater treatment

## Abstract

Zinc oxide nanoparticles (ZnO-NPs) possess unique properties, making them a popular material across various industries. However, traditional methods of synthesizing ZnO-NPs are associated with environmental and health risks due to the use of harmful chemicals. As a result, the development of eco-friendly manufacturing practices, such as green-synthesis methodologies, has gained momentum. Green synthesis of ZnO-NPs using biological substrates offers several advantages over conventional approaches, such as cost-effectiveness, simplicity of scaling up, and reduced environmental impact. While both dried dead and living biomasses can be used for synthesis, the extracellular mode is more commonly employed. Although several biological substrates have been successfully utilized for the green production of ZnO-NPs, large-scale production remains challenging due to the complexity of biological extracts. In addition, ZnO-NPs have significant potential for photocatalysis and adsorption in the remediation of industrial effluents. The ease of use, efficacy, quick oxidation, cost-effectiveness, and reduced synthesis of harmful byproducts make them a promising tool in this field. This review aims to describe the different biological substrate sources and technologies used in the green synthesis of ZnO-NPs and their impact on properties. Traditional synthesis methods using harmful chemicals limit their clinical field of use. However, the emergence of algae as a promising substrate for creating safe, biocompatible, non-toxic, economic, and ecological synthesis techniques is gaining momentum. Future research is required to explore the potential of other algae species for biogenic synthesis. Moreover, this review focuses on how green synthesis of ZnO-NPs using biological substrates offers a viable alternative to traditional methods. Moreover, the use of these nanoparticles for industrial-effluent remediation is a promising field for future research.

## 1. Introduction

Nanotechnology has revolutionized the world by enabling the production of materials with unique and superior properties. Nanoparticles, which are particles with at least one dimension less than 100 nanometers, are a class of materials that have gained significant attention attributed to their potential applications in several fields like agriculture, food processing and packaging, water treatment, aquaculture, pharmaceuticals, and cosmetics [1,2,3,4]. However, the traditional chemical methods used for green nanoparticle synthesis have several drawbacks, including the use of toxic chemicals, high energy consumption, and non-renewable resources. As a result, there is a growing interest in the development of eco-friendly and sustainable methods for the production of nanoparticles [5,6,7,8]. One such approach is the use of biological components as reducing and stabilizing agents in the green manufacturing of nanoparticles [9,10]. Nanoparticles, including carbon nanotubes, zerovalent nanoparticles, nanocomposites, and metal oxide nanoparticles, show great promise for use in different wastewater ecosystems [2,11].

Physical and chemical processes are the primary methods used for metal oxide nanoparticles to synthesize, but these technologies require the use of extremely hazardous and reactive-reducing substances, like sodium borohydride and hydrazine hydrate. In addition, using complex equipment, difficult processes, and harsh experimental conditions presents major challenges [12]. Physical methods tend to produce heterogeneous nanoparticles and require high energy inputs, while chemical methods use artificial capping, reducing, and stabilizing agents to produce the necessary homogenous metallic nanoparticles with less environmentally harmful byproducts [13]. However, nanoparticles produced using physical and chemical processes cannot be used for medical purposes due to their potential effects on human health [14]. The use of biological components such as microorganisms, extracts, and enzymes as reducing and stabilizing agents in the green synthesis of nanoparticles has gained significant attention in recent years [15]. The use of biological components offers several advantages over traditional chemical methods, including eco-friendliness, biocompatibility, and cost-effectiveness. Moreover, the use of biological components as reducing agents is a cheaper and eco-friendly alternative to traditional chemical-synthesis methods [16].

Zinc oxide nanoparticles (ZnO-NPs) are commercially important metal oxides due to their unique physical and chemical properties. ZnO-NPs have found numerous applications in various industrial domains, such as catalysis, solar cells, paints, UV light-emitting devices, electrical devices, biomedicine, and cosmetics [17]. The demand for ZnO-NPs is expected to increase in the coming years due to their potential applications in emerging fields such as nanoelectronics and nanomedicine. However, the traditional methods used for the ZnO-NPs synthesis have several limitations, such as high energy consumption, high cost, and environmental pollution [18].

Seaweed and marine microalgae have shown enormous potential for use in many biotechnological fields due to their numerous bioactive components [19,20,21]. Aquacultured marine macroalgae can be a sustainable source of biomass for the green production of metal oxide nanoparticles [22]. Several studies have reported the use of different species of seaweed and microalgae for the green synthesis of ZnO-NPs [7,23,24]. For example, *Sargassum* [25], *Caulerpa* [26], *Gracilaria* [27], *Pterocladia* [3], and *Arthrospira* [28] are commonly used for the synthesis of ZnO-NPs due to their high content of reducing agents and other bioactive compounds. Rhodophyta, in particular, has demonstrated potential for the green synthesis of ZnO-NPs due to the presence of unique bioactive components that enhance the reduction of metal ions and prevent the growth of nanoparticles [3]. Marine macroalgae have an advantage over terrestrial plants in the biosynthesis of ZnO-NPs because they do not compete for agricultural land or freshwater resources. As a result, they are a sustainable source for large-scale production of green nanoparticle synthesis [8,29,30].

The review by Agarwal et al. [31] discusses the various methods for the green synthesis of ZnO-NPs, including the use of plant extracts, microorganisms, and algae extracts. The review highlights the advantages of using green-synthesis methods, such as their low cost, eco-friendliness, and potential for large-scale production. Also, the review emphasizes the potential applications of green-synthesized ZnO-NPs in various fields, including wastewater treatment [31]. The study by Fouda et al. [32] explored the potential of using *Ulva fasciata* in the green synthesis of ZnO-NPs. The results indicated that the algae extract could successfully reduce zinc ions to ZnO-NPs in a fast and eco-friendly manner. The synthesized nanoparticles were characterized using various techniques, including SEM, XRD, and FTIR, confirming the formation of pure ZnO-NPs. The antibacterial activity of the synthesized ZnO-NPs was also evaluated, showing high inhibitory effects against gram-negative bacteria. Moreover, the ZnO-NPs were found to exhibit good photocatalytic activity towards the degradation of methylene blue, and they were also able to remove Cr(VI) ions from tanning wastewater. The study concluded that Ulva fasciata Delile extract could be a promising and sustainable source for the green synthesis of ZnO-NPs with potential applications in various fields, including antibacterial and photocatalytic activities and tanning-wastewater treatment [32].

The algal extracts act as stabilizing agents, preventing the agglomeration and growth of nanoparticles. The green synthesis of ZnO-NPs using algal extracts is a relatively simple and low-cost process that requires minimal technical expertise and equipment [33]. The green synthesis of ZnO-NPs using algal extracts has numerous industrial applications, including catalysis, solar cells, paints, UV light-emitting devices, electrical devices, biomedicine, and cosmetics [34]. Additionally, the use of ZnO-NPs in wastewater treatment has gained attention due to their ability to remove heavy metals, organic pollutants, and pathogens, making them a potential alternative to traditional wastewater-treatment methods. Furthermore, the synthesis of ZnO-NPs using algae extracts can be achieved through a simple, low-cost, and environmentally benign method [35]. The green synthesis of ZnO-NPs using algae extracts involves the use of algae extracts as a reducing agent to reduce the zinc ions to form nanoparticles and as a stabilizing agent to prevent the agglomeration of nanoparticles. The synthesis process is generally carried out at low temperatures, thereby making it more energy-efficient than traditional synthetic methods [36]. In addition to being a sustainable and environmentally friendly method, the use of seaweed extracts in the green synthesis of ZnO-NPs has numerous advantages. Seaweeds and marine microalgae contain various bioactive compounds such as polysaccharides, phenolic compounds, and pigments that can act as reducing and stabilizing agents in the synthesis of ZnO-NPs [3]. These compounds are not only abundant in seaweeds but are also cost-effective, readily available, and non-toxic [28]. Moreover, seaweed extracts have the advantage of being able to form nanoparticles of different sizes and shapes, depending on the extraction conditions, which can be tailored to suit specific applications [37].

This article review aims to promote green biochemistry through the easy, cheap, and environmentally benign green synthesis of ZnO-NPs using algal extracts, which is a relatively novel method and leads to reliable green chemistry, in addition to the application of those environmentally friendly metal oxide nanoparticles produced through synthetic means, especially in wastewater treatment.

## 2. Nanoparticles 

Nanomaterials (NMs) are objects with sizes between 1 and 100 nm, at minimum in one dimension, with unique characteristics in terms of porosity, size, shape, and other factors. Nanoparticles can be separated into various classes based on their size, shape, and chemical and physical characteristics. Some of them are categorized as metal NPs, ceramic NPs, polymeric NPs, carbon NPs, lipid NPs, and semiconductor NPs. In general, nanoparticles are highly complex entities and composed of three layers. The first layer is the surface layer, which can be functionalized with a wide range of small molecules, metal ions, surfactants, and polymers. The second layer is the shell layer, which is formed of a material that is completely different chemically from the core. Finally, the third layer is the core, which is the actual center of the nanoparticles. 

In general, there are two types of nanoparticles: organic nanoparticles and inorganic nanoparticles [38].

### 2.1. Organic Nanoparticles 

Organic nanoparticles are composed of either natural or synthetic carbon-based molecules or come in various categories, such as micelles, protein/peptides, liposomes, dendrimer-based nanoparticles, capsules, polymer conjugates, polymersomes, and polymeric NPs. Their properties, such as size, shape, and content, are greatly affected by these factors. While organic nanoparticles are generally safe, biocompatible, and biodegradable, they can be prone to degradation at high temperatures due to intracellular enzymes present in cells. Examples of organic NPs include Poly-L-lysine, quaternary ammonium compounds, cationic quaternary polyelectrolytes, N-halamine compounds, and algae and chitosan. Because of their instability at high temperatures, organic NPs are often preferred for their antibacterial properties [39].

### 2.2. Inorganic Nanoparticles

Inorganic nanoparticles are materials with nanometer-scale dimensions composed of non-carbon elements, including metals, metal oxides, and nonmetals. These nanomaterials possess unique physicochemical properties such as high surface area, high reactivity, and tunable–optical properties that make them suitable for a broad range of applications in various fields such as biomedicine, electronics, energy, and environmental remediation [40,41].

#### 2.2.1. Metal Nanoparticles

Metallic nanoparticles (M-NPs) are inorganic nanoparticles that have a metal or metal oxide core that is typically covered by an organic or inorganic material or metal oxide shell. M-NPs have numerous properties, such as mechanical hardness, large surface area, low melting point, and optical and magnetic characteristics. Metallic-nanoparticle-based catalysts are highly active and selective and have a long life for various chemical processes. Commonly studied M-NPs include silver, gold, copper, titanium, platinum, magnesium, and zinc NPs. Nanomaterials can be categorized based on the number of dimensions they possess at the nanoscale, including zero, one, two, and three dimensions. Nanoparticles, which have all dimensions less than 100 nm, belong to the zero-dimensional-nanomaterial category. Metal nanoparticles have received considerable attention from the scientific community worldwide owing to their distinctive surface-plasmon resonance and optical properties [42].

#### 2.2.2. Metal Oxide Nanoparticles

The reaction between a metal cation and oxygen gas results in the formation of metal oxides. Metal oxides (MOs) are an interesting class of inorganic compounds that have attracted much research and study due to their wide range of properties and characteristics. The useful magnetic, electrical, and optical properties of transition metal oxide nanoparticles make them a significant class of materials [43,44]. This makes them more suitable for a range of uses, including sensors, catalysis, batteries, lithium-ion, and environmental applications, among others. Transition metal oxides have a variety of fascinating properties that make them deserving of study in various applications when compared to other classes of materials [42]. The nature of metal oxides is more complex than that of pure metals, and their bonding can range from being almost ionic to being extremely covalent or even metallic. There are many different types of metal oxides, and each has its composition, morphology, structure, and physicochemical properties. Metal oxide nanoparticles, in particular, are of interest due to their significant and amazing optical, electrical, and magnetic properties [45]. Metal oxide nanoparticles (MONPs) are highly useful in a wide range of industrial activities, including sensors, electronics, solar energy conversion, catalytic processes, and magnetic-storage media. 

#### 2.2.3. Non-metallic Nanoparticles

Non-metallic nanoparticles are generated using non-metallic elements, which consist of various non-metal elements from distinct groups in the periodic table, including group IV elements (such as carbon and silicon) and group V elements (such as phosphorus). Doped nanoparticles, on the other hand, are often produced by incorporating Group V elements with other metals and nonmetals. Phosphate and phosphorus combine to generate ceramic nanoparticles. Nonmetal nanoparticles, for example, graphene, fullerenes, carbon nanotubes, and silica, have a distinctive structure and a large surface area, are biocompatible, have intriguing redox and catalytic capabilities, and are mechanically stable [46]. 

## 3. Green Synthesis of ZnO-NPs from Micro and Macroalgae

Algae are classified based on their cell walls’ chemistry, the presence of flagella, and other characteristics, with microalgae and macroalgae falling into different categories. However, the most widely used classification feature for algae is the presence of specific pigments other than chlorophyll. This helps in identifying macroalgae, which can be classified as green algae (Chlorophyta), brown algae (Phaeophyta), and red algae (Rhodophyta), the three primary classified types of algae. Microalgae, which are the most primitive and simply organized members of the plant kingdom, typically exist as cells measuring about 3–20 μm. They are found widely and are ubiquitous, with aquatic microalgae being discovered in various locations, such as hot springs and glacial ice flows. Several chemical components that can be extracted from macro- and microalgae include polysaccharides, vitamins, lipids, proteins, dietary fiber, antioxidants, minerals, and polyunsaturated fatty acids [47]. To give a brief example, among the potential chemical compounds effective in cancer treatments are some phytochemicals like carotenoids, pigment, scytonemin, several calothrixins, and phlorotannins [48]. 

### 3.1. Algal Extract Preparations

The algal extract is used to create nanoparticles in phyconanotechnology, which is a more recent field of nanoscience. Algal extracts are used because they are less poisonous, easy to handle, and can develop at low temperatures. Algal phytochemicals function as an efficient metal-reducing and capping agent to create a durable coating on the metal NPs in one-step production [49]. According to the other study by Yew et al. [50], the following is how algae can produce metal oxide nanoparticles: (a) Before being soaked in deionized water, the gathered seaweeds were cleaned under running water to get rid of any dirt, salt, or other foreign objects. (b) After being thoroughly washed, the algae or seaweed was powdered, and the suitably dried algae were steeped in deionized water for many hours. (c) Using filter paper, the final extract was purified, and (d) after that, the ZnO-NPs were created by adding a salt solution that contained metal ions.

### 3.2. Mechanisms for Synthesizing Nanoparticles Using Algae or Their Extracts

The algae or their extracts are used to create nanoparticles in phyconanotechnology, which is a more recent field of nanoscience [46]. Algal phytochemicals function as an efficient metal-reducing and capping agent to create a durable coating on the metal NPs in one-step production. Algae are a common and valuable source for the synthesis of metallic NPs due to their abundance and ease of accessibility as materials [49]. Algal extracts are a rich source of many bioactive compounds; these chemical compounds act as reducing and capping agents when nanoparticles are produced [51]. The two methods for the synthesis of metallic nanoparticles are intracellular and extracellular. Metal nanoparticles are created by the reduction of metal salts through bioactive substances released by algae cells in extract media. Intracellular synthesis is the result of an algal cell’s defensive mechanism against a stress condition produced by metals, which diverts the algal cells’ metabolic machinery to reduce the toxic effects of metals. The process of creating nanoparticles inside cells involves binding positively charged metal ions to the cell wall’s surface or the cytoplasm’s negatively charged protein or enzyme groups. When the trapped metal ions are reduced to form tiny nuclei, a variety of morphological nanoparticles are synthesized [52]. An intracellular enzyme found inside algal cells causes metal salts to be internally converted to nanoparticles. Likewise, the type of synthesis-related enzymes involved determines where nanoparticle synthesis takes place. However, since [53] demonstrated the bio-reduction of metal ions by biomolecules of green synthesis, it has been assumed that the surface chemistry of these reducing agent-specific functional groups like -C=O, -CHO, -COOH, -C-O-C, -OH, and -C=C- plays a significant part in the reduction of metal ions. The four steps of bioreduction are usually the activation phase, growth phase, nucleation phase, and termination phase. As a result of the precursor salt solution’s metal ions being reduced by extract-contained biomolecules during the first stage, known as the activation phase, nanoparticles begin to develop gradually. Second, the crystalline is decreased by biometabolites during the nucleation phase while growing on the metal nuclei.

### 3.3. Synthesis of Various ZnO-NPs by Marine Algae

The biotechnological developments that explain the production of ZnO-NPs using algae have been the focus of this section of the review. ZnO-NPs are an n-type semiconducting metal oxide with several applications in biomedical systems, cosmetics, rubber, electronics, and rubber products [54]. The physical and chemical parameters evaluated revealed that only *Sargassum myriocystum* algae could produce ZnO-NPs, such as the metal concentration, reaction time, pH, temperature, and extract concentration. The reduction and stabilization of the NPs (36 nm), which remained undetectable for 6 months and demonstrated the material’s great stability, were caused by the water-soluble pigments called fucoidans that are found in algae extract. Also, their research shows that soluble phytochemicals such as lipids, protein, ascorbic acid, alginic acid, carbohydrates, flavonoids, and mannitols in *Sargassum myriocystum* function as stabilizing and reducing agents. The artificial nanoparticles were available in a range of shapes, including spherical, triangular, hexagonal, rod-shaped, and rectangular varieties. 

The macroalga *Gracilaria edulis* aqueous extract was used in an experiment by Priyadharshini et al. [55] to create ZnO-NPs. The Zn^2+^ aqueous solution might be converted to Zn^0^ nanoparticles using the algae extract. The precursor solution for ZnO-NPs changed from white to reddish brown as a result of nanoparticle production. The rod-shaped nanoparticle capped with quinine combined boosted the cellular toxicity against the human PC_3_ malignant cell line. Because they are rod-shaped, zinc oxide nanoparticles can penetrate cells more effectively and cause cell death because they have a larger surface area for adhesion to cells. In the study by Francavilla et al. [56], an additional way of synthesizing ZnO-NPs was presented. In their technique, a reactive milling process uses agar made from red seaweed *Gracilaria gracilis* as a sacrifice template material. The zinc precursor Zn(NO_3_)_2_ was converted into a highly crystalline hexagonal wurtzite structure during milling, and the resulting nanoparticles have sizes between 18 and 50 nm. The porous ZnO-NPs were discovered to have good photocatalytic capabilities and could be employed to break down aqueous solutions of phenol after being calcined at 600 °C. Table 1 provides a summary of the numerous studies encountered regarding the synthesis of zinc oxide nanoparticles by marine algae that were examined by SEM analysis. 

According to Abdulwahid et al. [57], green synthesis of ZnO nanoparticles was found preliminary by color change during algal extract exposure to an aqueous solution of zinc ions. Originally, the synthesis of ZnO nanoparticles happened within 15 min, as evidenced by a shift in the color of the aqueous solution from yellowish brown to white. The intensity of the color shift was proportional to the time spent incubating. After 20 min, there is no discernible color change, suggesting that the reaction of ZnO nanoparticle production has reached saturation.

**Table 1:** Synthesis of zinc oxide nanoparticles using marine algae.

Algal Species	Size (nm) and Shape	Refs
*Sargassum muticum*	3 to 57, hexagonal	[58]
*Gracilaria gracilis*	18 to 50, hexagonal	[56]
*C. peltata, S. myriocystum*	spherical, radial, triangle, rod, rectangle	[54]
*Chlamydomonas reinhardtii*	21 nm- hexagonal wurtzite	[59]
*Sargassum wightii*	20–62 nm- spherical	[60]
*Ulva lactuca*	10–50- Sponge-like asym- metrical shaped	[22]
*Ulva fasciata*	77.81 nm- spherical	[61]
*Gracilaria edulis*	65–95- rod-shaped	[55]
*Sargassum muticum*	30–57 nm	[62]
*Agathosma betulina*	15.8 nm	[63]
*Gracilaria edulis*	66–95- Rod shaped	[64]
*Chlorella* sp.	20 to 50 nm	[65]
*Sargassum muticum*	--	[66]
*Oedogonium* sp.	--	[67]

### 3.4. Properties of ZnO-NPs 

The key benefits of employing a green way to obtain nanoparticles are that it is a low-cost and straightforward technique. Nevertheless, because of the small size and shape obtained, as well as the special features of the biological substrates used, green nanoparticle synthesis can improve the attributes of these nanomaterials [68]. The green production technique has been found to improve features such as antibacterial activity, photocatalytic efficacy, and biocompatibility in the case of ZnO-NPs [69,70,71]. As a result, green-produced ZnO-NPs have a high potential to replace conventional ZnO-NPs and be used in the construction of nanocomposites. Biosynthesized ZnO-NPs, for example, can be used to make nanocomposites for anticancer and antimicrobial coatings in the biomedical industry, as well as to improve dye degradation, to mention a few applications [72].

### 3.5. Characterization of Algae-Synthesized ZnO-NPs

To characterize a nanoparticle means to analyze and describe its properties that occur after synthesis. These properties include features like the particle’s shape, size, surface plasmon resonance, polydispersity, crystallinity, presence of capping agents, associated functional groups, and surface charge. Designing a nanomaterial with the necessary practical applications depends on all these physical and chemical properties [3,7,8]. The descriptions of the methods to determine the characteristics mentioned above are presented below [73].

#### 3.5.1. Macroscopic Methodologies

In terms of nanoparticles, macroscopic methods are those that do not require the use of difficult equipment. The characteristic bright color of metal nanoparticles is caused by surface-plasmon resonance. An easy-to-use visible signature for the creation of nanoparticles is the appearance of their distinctive bright hue. Consider the visual confirmation of the creation of zinc oxide nanoparticles by the emergence of dark brown, white, and brown colors [74]. To visually verify nanoparticle synthesis, different nanoparticles in solution are listed in Table 2 according to the color they exhibit.

#### 3.5.2. Microscopic Characterization

Various techniques for characterizing nanoparticles at a microscopic level include atomic force microscopy (AFM), scanning electron microscopy (SEM), transmission electron microscopy (TEM), field emission scanning electron microscopy (FESEM), and high-resolution transmission electron microscopy (HR-TEM) [81]. These methods are used to analyze the dimensions, morphology, and other topographical and surface characteristics of nanoparticles. These techniques are essential for studying the physical and chemical properties of nanoparticles. These methods provide detailed information about the size, shape, and surface characteristics of particles, which is critical for understanding their behavior and potential applications [73]. 

For AFM, this technique uses a small tip mounted on a cantilever to scan the surface of a sample. The tip interacts with the sample’s surface, and the resulting deflection of the cantilever is used to generate an image of the sample’s topography. AFM is particularly useful for studying the surface properties of nanoparticles and can provide information about their size, shape, and surface charge [82,83]. SEM uses a beam of electrons to scan the surface of a sample, creating a high-resolution image. This technique is particularly useful for studying the morphology and surface structure of nanoparticles. SEM can provide information about the size, shape, and surface characteristics of nanoparticles [84]. FESEM is a type of SEM that uses a high-voltage field to generate a focused beam of electrons. This technique can provide high-resolution images of the surface of nanoparticles and can also be used to analyze their composition and crystal structure [85]. The study by Raja and Saranya [86] investigated the biosynthesis and characterization of ZnO-NPs using marine-brown algae *Sargassum wightii* and their antimicrobial activity. The synthesized NPs were characterized using various analytical techniques, including FESEM, and evaluated for their antimicrobial activity against several pathogenic bacteria and fungi. FESEM images revealed that the ZnO-NPs were spherical in shape, with an average size of 52.32 nm. This study demonstrates the potential of using biosynthesized ZnO-NPs from marine-brown algae as a promising antimicrobial agent. 

TEM is a high-resolution imaging technique that uses a beam of electrons to pass through a thin sample. The resulting image provides information about the internal structure, size, and shape of nanoparticles. The study by Djearamane et al. [87] investigated the cytotoxic effects of ZnO-NPs on the *Arthrospira platensis*. The researchers synthesized ZnO-NPs and characterized them using various analytical techniques, including TEM, and evaluated their toxicity on *A. platensis* using the tetrazolium-based assay (TAM). The results showed that the synthesized ZnO-NPs had a significant cytotoxic effect on Spirulina platensis, with a decrease in cell viability observed at concentrations as low as 10 µg mL^−1^. TEM images revealed that the ZnO-NPs were internalized by the cells and caused damage to the cell membrane and organelles. This study highlights the potential environmental impact of ZnO-NPs on aquatic organisms and suggests the need for further studies to evaluate their safety before their widespread use [87]. HR-TEM is an advanced form of TEM that can provide higher-resolution images and more detailed structural information about nanoparticles. In HR-TEM, a highly focused beam of electrons is transmitted through a thin sample, which can be a single nanoparticle or a small cluster of nanoparticles. The electrons interact with the sample, and the resulting image provides information about the internal structure and atomic arrangement of the nanoparticles. HR-TEM is particularly useful for studying the crystal structure and defects of nanoparticles, as well as their size and shape. It can also provide information about the composition and chemical bonding of nanoparticles, which is essential for understanding their properties and behavior. HR-TEM is a valuable tool for both fundamental research and practical applications in fields such as materials science, nanotechnology, and biomedicine [88]. 

The study by Shariati et al. [89] explored the inhibitory effects of functionalized indium-doped zinc oxide nanoparticles (In-ZnO-NPs) on algal growth (*Chlorella vulgaris* and *Scenedesmus quadricauda*) for the preservation of adobe mud and earthen-made artworks under humid conditions. The researchers synthesized the NPs and characterized them using various analytical techniques, including SEM, FESEM, and HRTEM. The inhibitory effect of the NPs on algal growth was evaluated using the standard growth inhibition test (GIT). The results showed that the functionalized In-ZnO-NPs had significant inhibitory effects on algal growth, with a growth-inhibition rate of up to 100% at a concentration of 200 mg L^−1^. SEM, FESEM, and HRTEM images revealed that the NPs interacted with the algal cells, causing damage to their structure and function. This study suggests that functionalized In-ZnO-NPs have potential applications in the preservation of adobe mud and earthen-made artworks under humid conditions. The study by Al-Buriahi et al. [90] provides a review of sustainable approaches for the elimination of rhodamine B dye from textile wastewater using nanoparticle photocatalysts. The authors discuss the properties and mechanisms of various types of nanoparticle photocatalysts, including titanium dioxide (TiO_2_), zinc oxide (ZnO), and graphene oxide (GO), and their efficiency in removing rhodamine B from wastewater. The researchers also present SEM, FESEM, and HRTEM images to illustrate the morphology and size distribution of the synthesized nanoparticles. The review highlights the importance of developing sustainable and eco-friendly approaches for the treatment of textile wastewater to reduce its environmental impact. Overall, the study suggests that the use of nanoparticle photocatalysts shows promising potential as a sustainable approach for the elimination of rhodamine B from textile wastewater. The study by Ghosh et al. [91] focuses on the bioprospecting of novel algal species using nanobiotechnology. The authors discuss the potential of utilizing algae for the biosynthesis of nanoparticles and their applications in various fields, including biomedicine, agriculture, and the environment. The researchers also present SEM, FESEM, and HRTEM images to illustrate the morphology and size distribution of the synthesized nanoparticles. The study highlights the importance of exploring and utilizing the unique properties of different algal species for the synthesis of nanoparticles with diverse applications. The researchers suggest that the bioprospecting of novel algal species with nanobiotechnology has the potential to lead to the discovery of new, sustainable, and eco-friendly approaches for various applications. 

#### 3.5.3. UV-Vis Spectroscopy

The particle size, shape, interparticle characteristics, and kinds of nanoparticles can be determined by changes in absorbance or wavelength. The synthesis of nanoparticles in solutions containing algal extracts and metal salts can be observed with a UV-Vis spectrophotometer to determine these changes. Each metallic nanoparticle has a distinctive wavelength range that produces distinctive absorption spectra, and the metallic nanoparticle solutions are tested in the 190–1100 nm range. The interaction of metallic nanoparticles with light, which produces the surface plasmon resonance phenomenon, causes the absorbance to peak in the specified wavelength range [3,7,8,23,37,92]. The study by Mansour et al. [3] focused on the green synthesis of ZnO-NPs using red seaweed *Pterocladia capillacea* and their potential for eliminating organic toxic dyes from aqueous solutions. The nanoparticles were characterized using various techniques. The results showed that the synthesized nanoparticles had a size range of 20–35 nm and a hexagonal quartzite structure as determined by XRD and SEM analysis. The BET analysis revealed a surface area of 20.62 m^2^ g^−1^, indicating a moderate surface area-to-volume ratio. The UV-Vis analysis showed that the nanoparticles had a strong absorption in the visible region, suggesting their potential for visible light photocatalysis. The fluorescence lifetime spectrometer (FLS) analysis showed that the nanoparticles had fluorescence properties, indicating their potential for fluorescence-based applications. The thermogravimetric analysis (TGA) analysis showed that the nanoparticles had good thermal stability up to 400 °C. Overall, the synthesized ZnO-NPs using red seaweed exhibited promising potential for eliminating organic-toxic dyes from aqueous solutions, and the green-synthesis approach offers an eco-friendly and sustainable alternative to conventional methods.

#### 3.5.4. Fourier Transform Infrared Spectroscopy (FTIR)

The FTIR technique is utilized to obtain information about functional interactions, various types of interactions, and group bonding. In the process of synthesizing nanoparticles using algae, the functional groups in the algal solution that are responsible for reduction, capping, and stabilization are identified using FTIR. The range of wavenumbers for FTIR is typically from 4000 to 400 (1/cm), and a resolution of 4 (1/cm) is used to extract functional information. Moreover, surface chemistry is investigated, and several functional and bioactive groups that are attached to the surface of the resulting nanoparticles are identified. The study conducted by Anand and Suresh [93] investigated the use of *Sargassum wightii* extract as a low-cost sensitizer for ZnO photoanode-based dye-sensitized solar cells (DSSCs). The extract was used as a natural dye to sensitize the ZnO photoanode, and the resulting DSSCs were characterized using UV-Vis spectroscopy, BET analysis, and FTIR spectroscopy. The UV-Vis analysis showed the absorption peaks of the natural dye at wavelengths suitable for solar cell applications. The BET analysis revealed a high surface area of the ZnO photoanode, which indicated its potential for use in DSSCs. The FTIR analysis confirmed the presence of functional groups in the natural dye, which could be responsible for its sensitization effect. The results showed that the DSSCs sensitized with Sargassum wightii extract exhibited good photovoltaic performance with a high power conversion efficiency. The study concluded that Sargassum wightii extract could be used as a low-cost and eco-friendly sensitizer for ZnO photoanode-based DSSCs [93].

#### 3.5.5. Energy Dispersive X-ray (EDX)

Energy dispersive X-ray spectroscopy (EDX) is a powerful analytical technique that is commonly used to determine the elemental composition of materials, including nanoparticles. EDX works by detecting characteristic X-rays emitted by the elements within a sample when it is bombarded with high-energy X-rays. The energy of these X-rays is proportional to the atomic number of the element, allowing for the identification of the elements present in the sample. In the context of nanomaterials, EDX is particularly useful for characterizing the elemental composition of nanoparticles synthesized from natural sources [87]. The study of Asif et al. [94] investigated the biofabrication and characterization of the cyanobacteria Oscillatoria sp.-derived ZnO-NPs and compared their bioactivity with commercially synthesized NPs. UV-Vis spectroscopy showed an absorption peak of 370 nm for the synthesized NPs. FTIR analysis confirmed the involvement of functional groups in the biofabrication process. XRD analysis showed the crystalline nature of the synthesized NPs with a wurtzite structure. DLS showed the average size of the NPs as 20.2 nm. SEM-EDAX and TEM-SAED showed the morphological features of the NPs with elemental composition analysis. The zeta-potential value of the synthesized NPs was −25.8 mV, indicating their stability. The bioactivity study revealed the effectiveness of the cyanobacteria-derived NPs as antimicrobial agents.

#### 3.5.6. X-ray Diffraction (XRD) 

X-ray diffraction (XRD) is a widely used technique for characterizing the crystalline structure of materials, including nanocomposites. XRD provides information on the crystal phase, crystallite size, lattice parameters, and degree of crystallinity of the sample. It works by measuring the diffraction pattern produced when X-rays are scattered by the crystalline structure of the sample [95]. The study by Vasistha et al. [96] investigated the use of a microalga *Chlorosarcinopsis* sp. ZnO-NPs association for efficient nutrient removal and improved biodiesel application in sewage wastewater treatment. The characterization of the synthesized nanoparticles was carried out using UV-Vis, FTIR, XRD, DLS, scanning electron microscopy-energy dispersive X-ray (SEM-EDAX), transmission electron microscopy-selected area electron diffraction (TEM-SAED), and zeta-potential techniques. The results showed that the synthesized nanoparticles had an average size of 15–30 nm, with a zeta potential of −19.1 mV, indicating good stability. The XRD analysis revealed the formation of the ZnO crystalline phase. The SEM-EDAX analysis confirmed the presence of Zn and O elements in the synthesized nanoparticles. The integration of microalgae and ZnO-NPs resulted in efficient nutrient removal, with a removal efficiency of up to 98% for nitrogen and 87% for phosphorus. Additionally, the integrated approach led to improved biodiesel application, with a lipid content of up to 49%. Overall, the microalgae-ZnO-NPs association showed great potential for efficient sewage wastewater treatment and improved biodiesel production [96]. The study by Azizi et al. [58] investigated the green biosynthesis of ZnO-NPs by aqueous extract of brown-marine macroalga *S. muticum*. The characterization of the synthesized ZnO-NPs was performed using various techniques. The UV–Vis spectra showed a peak at 356 nm, indicating the ZnO-NPs formation. FTIR revealed the presence of functional groups in the bio-reduction and capping of ZnO-NPs. XRD analysis confirmed the formation of ZnO-NPs with a hexagonal wurtzite structure. DLS analysis showed that the ZnO-NPs had an average hydrodynamic size of 57.3 nm. SEM-EDAX analysis revealed the morphology and elemental composition of the ZnO-NPs. TEM-SAED analysis confirmed the crystalline nature of the ZnO-NPs. Zeta potential analysis showed the stability of the synthesized ZnO-NPs. Overall, the study demonstrates the potential of *S. muticum* as a green and eco-friendly source for the biosynthesis of ZnO-NPs with potential applications in various fields, including medicine and agriculture [58].

#### 3.5.7. Raman Spectroscopy

Raman spectroscopy is a technique that can provide valuable information about the crystal structure, isotope composition, bimolecular interactions, and non-covalent molecular interactions of nanomaterials, including those derived from algae. This technique works by analyzing the monochromatic light or laser beam on the sample and measuring the dispersed light of the angle of the incident light while minimizing Rayleigh scatter. The photodetector can then measure the inelastically dispersed light at lower or higher frequencies. The resulting spectrum between the Raman shift and Raman’s intensity can provide useful information about the sample [97]. Zhang et al. [98] investigated the contribution of physicochemical transformations to the toxicity of aged ZnO-NPs to *C. vulgaris*. The physicochemical transformations were characterized using various techniques. The results showed that the aged ZnO-NPs had increased agglomeration, larger size, and reduced surface area as determined by HR-SEM and BET analysis. The XRD analysis showed that the crystal structure of the ZnO-NPs remained unchanged after aging. The FT-IR analysis showed the presence of new functional groups on the surface of the aged ZnO-NPs, indicating surface oxidation. The UV-Vis analysis showed a decreased absorption intensity of the aged ZnO-NPs in the visible region, suggesting reduced photocatalytic activity. The FLS analysis showed a red shift in the fluorescence spectra of the aged ZnO-NPs, indicating changes in the electronic structure. The TGA analysis showed that the aged ZnO-NPs had reduced thermal stability compared to fresh NPs. The results suggest that physicochemical transformations during aging play a crucial role in the toxicity of ZnO-NPs to *Chlorella vulgaris*. This study provides new insights into the variation of toxicity of ZnO-NPs under the aging process and highlights the importance of understanding the physicochemical transformations that occur over time.

#### 3.5.8. Potential Zeta

The net charge of a nanoparticle or other altered object can be determined using nanopowder by measuring the zeta potential of a solution. This reveals the stability of nanoparticles. The conventional method for determining a sample’s potential is to apply an electric field to it while monitoring how quickly charged species move toward an electrode. This is appropriate, given the possibility. A potential >30 mV indicates stability, but a potential of 30 mV indicates particles that appear to aggregate or to be unstable. The potential may be influenced by several factors, including pH, temperature, concentration, radiation, ionic solution strength, and surface ligands, with potential effects on nanoparticle stability as a result [99]. The study by Zeng, Chen, Zhu, and Yu [43] investigated the effect of nanocellulose on the dispersion and toxicity of ZnO-NPs to the green algae *Eremosphaera viridis*. The ZnO-NPs were characterized using various techniques. The results showed that the ZnO-NPs had a size range of 50–70 nm and a hexagonal wurtzite structure as determined by XRD and HR-SEM analysis. The BET analysis revealed a surface area of 13.7 m^2^ g^−1^, indicating a relatively low surface area-to-volume ratio. The FTIR analysis showed the presence of functional groups on the surface of the ZnO-NPs, indicating their interaction with the nanocellulose. The UV-Vis analysis showed a peak at 376 nm, indicating the presence of ZnO-NPs in the sample. The FLS analysis showed that the ZnO-NPs had fluorescence properties, indicating their potential for fluorescence-based applications. The TGA analysis showed that the ZnO-NPs had good thermal stability up to 500 °C. The Potential zeta analysis showed that the addition of nanocellulose increased the negative surface charge, indicating improved dispersion of the ZnO-NPs in the aqueous solution. The results showed that the addition of nanocellulose increased the toxicity of the ZnO-Ps to *Eremosphaera viridis*, which may be attributed to improved dispersion and increased uptake of the NPs by the algae. This study provides new insights into the effect of nanocellulose on the dispersion and toxicity of ZnO-NPs to green algae and highlights the potential risks associated with the use of nanocellulose in environmental and biotechnological applications [43].

#### 3.5.9. Thermo-Gravimetric Analysis (TGA)

TGA is a method that measures the mass change in a sample, and it is used to detect evaporation, decomposition, oxidation, and other effects of temperature change that cause mass changes. The study by Pandimurugan and Thambidurai [100] investigated the use of seaweed-capped (*Padina tetrastromatica*) ZnO-NPs for the photodegradation of dyes and antibacterial activity. The synthesized nanoparticles were characterized using various techniques. The size of the synthesized seaweed-capped ZnO-NPs was found to be in the range of 15–25 nm, as determined by HR-SEM analysis. The XRD analysis showed that the nanoparticles had a hexagonal quartzite structure. The BET analysis revealed that the surface area of the seaweed-capped ZnO-NPs was 35.8 m^2^ g^−1^, indicating a high surface area-to-volume ratio. The TGA analysis showed that the nanoparticles had good thermal stability up to 300 °C. The UV-Vis analysis showed that the seaweed-capped ZnO-NPs had a high absorption in the visible region, indicating their potential for visible light photocatalysis. The FLS analysis showed that the ZnO-NPs had a broad emission peak in the visible region, indicating their potential for fluorescence-based applications. The results indicate that the seaweed-capped ZnO-NPs have a smaller size, higher surface area, and greater photocatalytic activity compared to bare ZnO-NPs. The nanoparticles also showed improved antibacterial activities against both gram-negative and -positive bacteria. Overall, the seaweed-capped ZnO-NPs exhibit promising potential for environmental and biomedical applications [100]. The study by Chen et al. [101] investigated the adaptive interactions between ZnO-NPs and *Chlorella* sp, which were characterized using various techniques. The results showed that the ZnO-NPs had a size range of 20–30 nm and a hexagonal wurtzite structure as determined by XRD and HR-SEM analysis. The BET analysis revealed a surface area of 40.2 m^2^ g^−1^, indicating a high surface area-to-volume ratio. The FTIR analysis showed that the ZnO-NPs had interactions with functional groups on the surface of the algae, indicating their ability to adsorb onto the algae surface. The UV-Vis analysis showed that the ZnO-NPs had a high absorption in the visible region, suggesting their potential for visible light photocatalysis. The FLS analysis showed that the ZnO-NPs had fluorescence properties, indicating their potential for fluorescence-based applications. The TGA analysis showed that the ZnO-NPs had good thermal stability up to 500 °C. The results suggest that the adaptive interactions between ZnO-NPs and *Chlorella* sp. are influenced by the physicochemical properties of the NPs and the *Chlorella*. This study provides new insights into the adaptive interactions between ZnO-NPs and algae and highlights their potential for environmental and biotechnological applications.

## 4. Application of ZnO-NPs for Wastewater Treatment

### 4.1. Adsorption Technique for the Removal of Different Pollutants

The most common techniques for treating polluted liquids are adsorption techniques. This technique for removing organic and inorganic pollutants involves the accumulation of metal ions onto adsorbent pores and surfaces, resulting in a layer that contains ions as well as other contaminants. Adsorption is preferred over conventional metal removal techniques due to a number of its advantages: (1) Economical: adsorbents are inexpensive. (2) Metal selection: the metal-sorbing performance of various kinds of biomass can be more or less on various metals. (3) Regenerative: adsorbent can be reused after the metal has been recycled. (4) Uses less energy as well as chemical consumption. (5) No production of sludge. (6) There may be metal recovery, and (7) competitive performance: it can be performance comparable [72]. Factors that influence the adsorption efficiency comprise the amount of adsorbent, adsorbate–adsorbent interaction, temperature, adsorbent surface area, contact time, particle size, pH, initial pollutant concentration, etc. [92].

#### 4.1.1. Removal of Inorganic Pollutants by Adsorption Technique 

Cr (III), Fe (II), Ni (II), Cu (II), Zn (II), Hg (II), and Pb are harmful toxic heavy metal effluent that is released into the environment by a variety of companies including those that perform mining operations, metal plating, and battery production (II). They do not biodegrade and tend to build up in aquatic environments. When the concentration of these metal ions exceeds the permissible level set by the World Health Organization (WHO) and the United States Environmental Protection Agency, they are considered contaminants (USEPA). Due to the serious health and environmental dangers they provide, it is essential to find new and effective adsorbents and strategies to remove them from water and wastewater. Nonetheless, few studies have been carried out on the application of green ZnO-NPs by algae for the removal of inorganic pollutants by adsorption technique [102,103]. Table 3 summarizes some applications of green ZnO-NPs for the removal of inorganic pollutants.

Fouda et al. [108] studied the ZnO-NPs to test their effectiveness in the removal of Cr (VI) from an aqueous solution. This study showed that 93.4% of the total Cr (VI) was removed when the tanning effluent was treated with ZnO-NPs. ZnO-NPs from *Oedogonium* sp. algae were investigated by Khilji et al. [67] for their capacity to remove heavy metals from wastewater used in the leather industry. The leather effluents had greater concentrations of Cr (310.1), Cd (210.5), and Pb (75.5 mg L^−1^) before the addition of nanoparticles. After 45 days of treatment with 1 mg of nanoparticles, the removal efficiencies of TDS, chlorides, Cr, Cd, and Pb were increased by 46.5%, 43.5%, 54%, 57.6%, and 59.3%, respectively. In the study by Vasistha and Rai [96], unique ZnO-NPs were used to integrate the microalga *Chlorosarcinopsis* sp. with ZnO-NPs for the treatment of wastewater. The *Chlorosarcinopsis* sp. microalgae were grown in primary-treated wastewater and mixed with ZnO-NPs in secondary-treated wastewater to treat sewage wastewater and produce high-quality biodiesel. When the microalgae were cultivated in primary-treated wastewater and combined with 10 mg L^−1^ ZnO-NPs, they achieved a removal efficiency of 87.20%, 97.5%, and 82.21% for total nitrogen, total organic carbon, and total phosphorus, respectively [96].

#### 4.1.2. Removal of Organic Dyes by Adsorption Technique 

Nanoadsorbents are useful for removing metal ions and organic molecules, and functionalization can improve their selectivity for particular contaminants [19]. Table 3 demonstrates that ZnO-NPs are effective at eliminating organic contaminants and that functionalization can raise their selectivity for a specific pollutant. Nanoscale zinc oxide has been investigated as an inexpensive, efficient adsorbent for water treatment [109]. However, the application of green zinc oxide nanoparticles by algae for water treatment has only been covered in a few academic reviews. Hassaan et al. [104] studied the Congo red dye removal and antibacterial activity of both green and chemically produced ZnO-NPs. Algae from the *Gelidium pulchellum* genus produce green ZnO-NPs. According to the report’s results, after the first three minutes of contact with ZnO-NPs, 85% of the Congo red (CR) dye had been decolored. The decolorization of Congo red dye increases slowly and reaches a higher removal of 100% using 0.1 g of ZnO-NPs 10 mL at pH 6, and 50 mg L^−1^ dye concentration at ambient temperature. The greenly synthesized ZnO-NPs lead to 100% Congo red dye removal for 25 and 50 mg L^−1^ dye concentrations. Mansour et al. [3] investigated ZnO-NPs synthesized from the red seaweed (*Pterocladia capillacea*), and their ability to remove organic hazardous dye (isomeric violet 2R) ions from an aqueous solution will be assessed. Also, 0.08 g of ZnO-NPs were applied at a temperature of 55 °C, a pH of 6, and a contact time of 120 min with a 99% removal of Ismate violet 2R. The ZnO-NPs have a dye-adsorption capability of 72.24 mg g^−1^. 

#### 4.1.3. Treatment of Oil and Hydrocarbon

Only few research has been done on the use of green metal oxide nanoparticles by algae for the adsorption technique’s removal of hydrocarbon and oil contaminants [110]. The study by Hassan et al. [111] investigated the use of iron oxide nanoparticles made via green synthesis for the elimination of pyrene and benzo (a) pyrene (PAHs) micropollutant from water. The adsorption factors were observed. These variables include the dose of nanoparticles, pH, temperature, and initial PAH concentration. According to this study, the results showed that green iron oxide nanoparticles have maximal-adsorption capacity of 2.8 and 0.029 mg g^−1^, for both pyrene and benzo (a) pyrene, respectively. In another study, green iron nanoparticles with a spherical form and a size range of 5–10 nm were created for hydrocarbon treatment. The ability of the biosynthesized iron nanoparticles to remove total petroleum hydrocarbons (TPH) from contaminated soil and water was investigated. A total of 88.24% TPH was removed from the water sample by the iron nanoparticles after 12 and 32 h of treatment, respectively [111].

#### 4.1.4. Water Disinfection from Microbes

Algae have a wide variety of polyphenolic chemicals as the importance of their antioxidative characteristics. The production of nanoparticles is made non-toxic and environmentally friendly by using microalgae extracts as capping and reducing agents. The effectiveness of magnetic NPs treated with polyallylamine hydrochloride (PAAH) in removing harmful germs from drinking water through electrostatic interaction and magnet capture was investigated. *Escherichia coli*, Acinetobacter, Pseudomonas, and Bacillus were the four main pathogenic species with high removal efficiencies. The study indicated high removal efficiency (99.48%) of bacteria and total bacteria residual counts as low as 78 CFU mL^−1^, which exceeded the WHO drinking water criterion of 100 CFU mL^−1^ [112].

### 4.2. Photocatalytic Applications of ZnO-NPs for The Removal of Pollutants

#### 4.2.1. Photocatalysis and Its Importance

The terms “photo” and “catalysis” are the origin of the term “photocatalysis.” “Photo” stands for light, and “catalysis” is the process of changing a chemical reaction while a catalyst is present. Similar to semiconductors, photocatalysts can oxidize organic compounds when they are exposed to visible sunlight [113]. Photocatalysis is a promising technique for the degradation of organic pollutants in wastewater. For a photocatalyst to be effective, the band-gap energy must be less than 3 eV to extend the light absorption into the visible region. In addition, the photocatalytic process can occur either homogeneously or heterogeneously depending on the physical states of the catalyst and interacting species. In homogeneous photocatalysis, all reactive species are in the liquid, solid, or gaseous states of matter [114]. Heterogeneous photocatalysis occurs when the reactive species exist in many states or various physical states. A solid photocatalyst typically comes into contact with contaminants that are either aqueous or gaseous. Thus, a photocatalyst is referred to as a surface catalyst because it gives the surface—active sites that interact with the reactant and causes the chemical reaction to occur. For heterogeneous photocatalysis, semiconductors are usually favored over other photocatalysts. These substances have high activity, are poison- and defections, recyclable, chemically and mechanically stable, economical, chemically and biologically inert, and nonselective under many conditions. According to specific reports in the literature, wastewater pollutant degradation using MONPs is possible. ZnO-NPs are present in the most significant metal oxide nano photocatalyst [115].

#### 4.2.2. The Characteristics of Photocatalytic Substances

A catalyst must be able to use light energy (rather than thermal energy) to catalyze the chemical process in order to act as a photocatalyst. Depending on the concerned reaction, which only occurs in the presence of light, it could be a semiconductor, an organic compound, or a coordination compound. It showed how nano-photocatalysts could increase oxidation capacity due to the strong production of oxidizing species at the substance’s surface, which effectively assists in the degradation of pollutants from contaminated water [116]. 

Photocatalytic degradation is influenced by various factors, including the amount of catalyst and pollutant, the pH of the solution, temperature, time, and morphology of the photocatalyst. The amount of photocatalyst plays a crucial role in photocatalytic degradation, where an optimum amount of catalyst is required for maximum degradation efficiency [117]. Similarly, the amount of pollutant also affects the degradation efficiency, where higher pollutant concentration can hinder the degradation process. The pH of the solution also affects photocatalytic degradation, as it can alter the surface charge of the photocatalyst and the adsorption behavior of the pollutant [118]. The effect of temperature on photocatalytic degradation is linked to the kinetic energy of the reacting species, where higher temperature can increase the reaction rate but may also lead to catalyst deactivation. The duration of irradiation also affects photocatalytic degradation, where longer irradiation time can lead to higher degradation efficiency. Additionally, the morphology of the photocatalyst can also affect the photocatalytic degradation efficiency, where materials with high surface area, narrow bandgap, and special morphologies such as nanowires and nanotubes have been reported to have superior photocatalytic activity [119]. 

#### 4.2.3. Photocatalytic Technique for Removal of Organic and Inorganic Pollutants

When a catalyst, typically a semiconductor oxide, is activated by ultraviolet (UV) or visible irradiation, a chemical reaction (oxidation or reduction) is accelerated more quickly. This process is known as photocatalysis. A chemical change can only be initiated or accelerated with the help of light and a catalyst. Wastewater contains a lot of organic contaminants, which are particularly bad for both aquatic and terrestrial ecosystems. Examples of these pollutants include textile dyes, pesticides, and pharmaceutical waste. The ZnO-NPs’ excellent chemical and physical characteristics, such as their high chemical stability and low cytotoxicity, make them suitable materials for photocatalytic experiments [111]. ZnO-NPs is the chemical that is most commonly used to study photocatalyst activity in comparison to other chemicals [111]. With a binding energy of 60 MeV, zinc oxide is a fantastic wide-bandgap, natural n-type semiconducting material that is abundant in nature, nontoxic, cheap cost, and environmentally beneficial photocatalyst. It is more suitable for applications involving dye-sensitive solar cells and solar photovoltaic systems because it can absorb a wider spectrum of energy. 

Dehghani and Mahdavi [120] studied ZnO-NPs and UV irradiation to remove the acid dye 4092 from an aqueous solution. The dye samples were exposed to UV irradiation for 2 to 12 min. Studies revealed that the treatment of dye was achieved at 0.5 mg L^−1^ of dye concentration, 12 min of radiation period, pH 5, and 0.2 g L^−1^ of catalyst dose. Moreover, reactive blue dye is degraded by a CdO-ZnO nanoparticle, which is produced from macroalgae on seaweed. Furthermore, malachite green dye degrades quickly in *Sargassum* species supporting ZnO-NPs and Co-ZnO composites when exposed to visible light. Reactive blue 198 dye is degraded by a CdO-ZnO nanoparticle from macro algae when exposed to visible light, UV light, and direct sunlight. The holes make hydroxyl radicals when they react with water molecules, while photogenerated electrons generate superoxide radicals when they interact with oxygen. Dye degradation is caused by these reactive species [106].

Gu et al. [121] investigated the phycosynthesis and enhanced photocatalytic capabilities of zinc oxide nanoparticles for removing organosulfur pollutants. They utilized Chlorella sp. microalgae extract to create ZnO-NPs that were safer, more stable, and of higher purity using an environmentally sustainable approach. Microalgae *chlorella* extract is used for the first time as a natural nanofactory for ZnO-NP biosynthesis. By comparing the retention times of the DBT sample that was prepared in the lab and its final photocatalyzed solution by ZnO-NPs, the reduction of DBT concentration was identified in the gas chromatograph (GC). After 3 h of photocatalytic reaction, 97% of the DBT pollutant was destroyed, according to the qualitative analysis. By utilizing a green ZnO nanophotocatalyst, degradation of the organosulfur model contaminant Dibenzothiophene (DBT) was achieved at a rate of 97% under mild conditions and neutral pH. The nanophotocatalyst exhibited high efficiency and durability, as it could be easily separated and recycled for up to five consecutive runs. These green ZnO-NPs were synthesized and utilized as a nanocatalyst for treating sewage from petroleum refineries. The ZnO-NPs at a concentration of 1 g L^−1^ were utilized as a photocatalyst for 4 h at 30 °C under UV light. For the polycyclic aromatic hydrocarbon acenaphthylene, the optimum elimination rate was 73% [122].

#### 4.2.4. General Reaction Mechanism of Photocatalysis

Various groups have already discussed the fundamental mechanisms involved in the photocatalytic degradation of pollutants. The main function of a photocatalyst in this process is to expedite the various oxidation and reduction reactions that take place in the presence of light, according to a review paper by Theerthagiri et al. [123]. The photocatalytic degradation of pollutants typically involves three general steps: firstly, upon light irradiation, the separation of hole and electron occurs; secondly, the charge carriers scatter on the surface of the photocatalyst; and lastly, the light-driven catalytic oxidation and reduction reactions take place on the active sites of the catalyst. This process has significant potential for addressing environmental pollution and is an area of active research and development [117].

#### 4.2.5. Mechanisms for Heavy Metal Ion Elimination by ZnO Particles

Two methods for heavy metal ion removal by ZnO particles are proposed: (a) physical adsorption and (b) reduction/oxidation by photo-generated electron-hole pairs.

##### Adsorption by Physical Means

According to Thein et al. [124], the negatively charged surface of ZnO particles was primarily contributed by the OH^−^ groups throughout the development phase. These OH^−^ groups were transformed into actively adsorptive sites. Heavy metal cationic in an aqueous solution reacted with OH^−^ groups to form a thin coating on the surface of ZnO particles. Wang et al. [125] observed a similar observation. Because the adsorption process was restricted by the number of negative adsorptive sites on the surface of ZnO particles, the adsorption efficiency was usually weak and could reach saturation after some time. Because the optical energy of visible light was insufficient to allow ZnO particles to create electrons and holes, metal ions were removed from the solution by ZnO particles through a physical adsorption process.

##### Photogenerated Electron-Hole Pairs Reduce/Oxidize

When the metal’s redox potential is greater than the (electron)_CB_ level of ZnO particles, the reduction occurs. Metal ions that are likely to be reduced are Ag(I) ions, Cr(VI) ions, and Cu(II) ions. Metal ion oxidation occurs selectively when the oxidation potential is less positive than the (hole)_VB_ level. Oxidation happened at the Pb(II) and Mn(II) ions, according to the data. It is worth noting that many electrons and holes could be created constantly for the reduction or oxidation of metal ions as long as the right optical-excitation source is available. As a result, the metals/metal oxides could exist as a thin layer on the surface of ZnO particles or as particles deposited on the surface of ZnO particles or in the solution [126].

In summary, heavy metal ions were removed by ZnO particles using one of the above-mentioned mechanisms or a combination of mechanisms depending on the metal ions and light sources used.

## 5. Conclusions and Future Perspectives

Zinc oxide nanoparticles (ZnO-NPs) have seen a significant surge in usage across various applications, including industrial catalysts, gas sensors, electronic materials, biomedicines, and environmental remediation. This exponential growth can be attributed to their exceptional properties, notably their high surface area-to-volume ratio, which provides them with unparalleled flexibility in application. Many studies have reported the possibility of obtaining ZnO-NPs through a green-synthesis process using a variety of algae. These substrates act as reducing and stabilizing agents or chelating substances despite their source. The green synthesis of ZnO-NPs using algae extracts is a promising method for the production of sustainable and environmentally friendly nanoparticles. Seaweeds and marine microalgae are abundant sources of biomass that can be used for the synthesis of ZnO-NPs, and the use of seaweed extracts as reducing and stabilizing agents is a low-cost, simple, and efficient method. Moreover, the green synthesis of ZnO-NPs using seaweed extracts has numerous advantages, such as the ability to produce nanoparticles of different sizes and shapes, the use of non-toxic and cost-effective bioactive compounds, and various applications in different fields such as biomedicine, wastewater treatment, and food packaging. Therefore, further research and development of this method are required to optimize the synthesis process and explore its full potential in various industrial domains. Additionally, researchers should investigate how nanoparticles’ size and shape can affect their properties and applications. By addressing these issues, ZnO-NPs can provide a promising solution for environmental remediation and other applications.

## Figures and Tables

**Table 2:** Characterization of specific ZnONPs produced through biological means.

Visible Confirmatory	UV–Vis Abs (Visible Peak nm)	FTIR Spectrum (cm^−1^)	Size (nm)	Band Gap Energy (eV)	Refs
White precipitate	377	393	22	3.29	[75]
	353	410	10	3.34	[76]
	377	429–490	9.6–25.5	3.87	[77]
	322–334	530	50–90	3.38	[78]
	400	445	20–30	3.46	[79]
	378	400–600	10	–	[80]

**Table 3:** Metal oxide-based nanomaterials for removal of organic and inorganic pollutants in wastewater.

Algal Species	Adsorbate/Pollutants	Optimization Condition	Method/Efficiency/Adsorption Capacity (mg g^−1^) or Removal (%)	Refs
*Pterocladia Capillacea*	Ismate Violet 2R	60–120 min, pH 2	59.88 %	[3]
*Gelidium pulchellum*	Congo red	20 min, pH 6	85%	[104]
*Oedogonium* sp.	Pb	1 g, 45 d	59.33 %	[67]
*Oedogonium* sp.	Cd	1 g, 45 d	54.04%	[67]
*Oedogonium* sp.	Cr	1 g, 45 d	53.60%	[67]
*Oedogonium* sp.	COD	1 g, 45 d	54.01%	[67]
*Oedogonium* sp.	BOD	1 g, 45 d	57.67%	[67]
*Calotropis procera*	Cd(II)	pH 6, 20 min, 0.4 g	84.75	[105]
*Calotropis procera*	Fe(III)	pH 6, 20 min, 0.4 g	126.32 mg g^−1^	[105]
*Chlorosarcinopsis *sp.	Total organic carbon	10 mg L^−1^	97.5%	[96]
*Chlorosarcinopsis *sp.	Total nitrogen	10 mg L^−1^	87.20%	[96]
*Chlorosarcinopsis *sp.	Total phosphorous	10 mg L^−1^	82.21%	[96]
*Padina gymnospora*	Reactive blue 198 dye	15 min	99.57%	[106]
*Ulva lactuca*	Methylene blue (MB)	120 min	90%	[22]
*Sargassum muticum*	Methylene blue	60 min	96%	[66]
*Chlorella species*	Dibenzothiophene	3 h, 25 °C, pH 7	97%	[107]
*Ulva fasciata*	MB	140 min, 35 °C, pH 7	84.9%	[32]
*Ulva fasciata*	COD	15 min	89%	[32]
*Ulva fasciata*	BOD	15 min	89%	[32]
*Ulva fasciata*	TSS	15 min	97%	[32]
*Ulva fasciata*	Conductivity	15 min	92	[32]
*Ulva fasciata*	Cr (VI)	15 min	93%	[32]

## Data Availability

Not applicable.
